# Mechanically Active Contractile Hydrogels for Skin Wound Repair

**DOI:** 10.3390/gels12080701

**Published:** 2026-08-05

**Authors:** Shang Chen, Shengkai Yu, Jiashuo Fan, Hua Zhang

**Affiliations:** Health Science Center, Ningbo University, Ningbo 315211, China; ichenpi2004@gmail.com (S.C.); yushengkai1996@outlook.com (S.Y.); 15158350991@163.com (J.F.)

**Keywords:** contractile hydrogel, wound repair, biomechanical cues, mechanotransduction

## Abstract

Polymer hydrogels have been widely explored for wound repair, yet conventional designs remain passive barriers with limited mechanical intervention. Contractile hydrogels address this gap by undergoing network densification and macroscopic shrinkage, which can be converted into wound-edge traction through interfacial adhesion. This review classifies contractile hydrogels into temperature-responsive, pH-regulated, intermolecular-interaction-driven, and solvent-mediated systems according to their dominant contraction mechanisms. The transduction of contraction-derived mechanical cues into biochemical signals is discussed across tissue, cellular, and molecular scales, with emphasis on the well-supported integrin/focal adhesion kinase (FAK)-associated focal adhesion pathway and mechanosensitive ion channels. Current applications in acute full-thickness defects, infected and diabetic chronic wounds, surgical incisions, and scar control are critically surveyed. Finally, key challenges pertaining to force transmission efficiency, spatiotemporal controllability, biosafety, and clinical translatability are discussed. This review aims to provide design guidelines for the rational development of contractile hydrogel platforms for advanced wound management.

## 1. Introduction

Skin is the primary barrier against mechanical injury, microbial invasion, and water loss. It is thus vulnerable to damage from trauma, burns, surgery, infection, or metabolic disease [1,2,3,4]. While small superficial wounds often heal spontaneously, large full-thickness defects, diabetic chronic wounds, infected wounds, and wounds located at mobile anatomical sites frequently heal poorly. In such cases, persistent inflammation, excessive reactive oxygen species, bacterial biofilms, insufficient angiogenesis, impaired cell migration, and disordered extracellular matrix remodeling result in a prolonged inflammatory or inefficient proliferative state of the wound [5,6,7]. Moreover, injury releases pre-existing skin tension, causing wound edges to retract, enlarging the defect, and exposing tissue to abnormal mechanical loading. Sustained mechanical imbalance may overactivate fibroblasts, dysregulate collagen deposition, and lead to hypertrophic scarring, contracture, or functional impairment [8,9]. Thus, wound repair is not merely a biochemical filling process, but a geometry- and force-dependent tissue remodeling process.

Polymer hydrogels are an attractive wound dressing due to their high water content, soft mechanical properties, exudate absorption capacity, and ability to deliver drugs or growth factors while maintaining a moist protective interface on the wound surface [10]. By incorporating functional moieties, hydrogels can be further endowed with multiple therapeutic activities, including antibacterial, anti-inflammatory, antioxidant, pro-angiogenic, and pro-epithelialization effects [11]. Nonetheless, most hydrogels still function primarily as passive coverings. When wound edges remain separated, irregular cavities persist in the wound, the material–tissue interface is repeatedly disrupted by body motion, or surrounding skin tension drives scar formation, passive coverage alone cannot adequately address the underlying mechanical challenges [12].

Numerous studies have demonstrated that mechanical signals are tightly coupled to cell behavior during wound repair [13,14,15]. Wound contraction is a normal healing process in embryonic wounds, where actin cables generate purse-string-like centripetal forces that rapidly draw wound edges together. However, adult wounds rely more heavily on inflammation, fibroblast migration, myofibroblast contraction, and extracellular matrix remodeling [16]. Moderate mechanical traction supports cell migration, proliferation, and angiogenesis, whereas sustained excessive tension may promote fibrotic responses through signaling networks involving integrin/FAK, phosphoinositide 3-kinase (PI3K)/protein kinase B(Akt), RhoA/Rho-associated coiled-coil-containing protein kinase (ROCK), Yes-associated protein (YAP)/transcriptional coactivator with PDZ-binding motif (TAZ), Piezo/Ca^2+^, and transforming growth factor-β1 (TGF-β1)/SMAD pathways [17]. A desirable wound dressing for mechanical therapy should therefore provide traction within an appropriate temporal and spatial window. Such traction must be sufficient to promote closure, yet not so prolonged or intense as to amplify pathological scarring.

Contractile hydrogels represent an emerging class of mechanically active wound dressings. Their efficacy does not rely on simple volume shrinkage, but on the transformation of internal network rearrangements into usable mechanical forces at wound interfaces. Such structural changes include hydrogel deswelling, polymer chain collapse, molecular aggregation, and secondary structure rearrangement. Through wet adhesion, edge fixation, and structural constraint, hydrogel contraction generates centripetal traction. This effect reduces wound opening, promotes the approximation of wound edges, eliminates residual dead space under conformal contact, and remodels local tissue tension. Different hydrogel systems achieve contractile behavior via distinct mechanisms. Thermoresponsive hydrogels contract through polymer chain dehydration and collapse above their lower critical solution temperature. pH- and ion-responsive hydrogels undergo contraction driven by variations in surface charge density, osmotic pressure and ionic complexation. Multiple molecular interactions, including hydrogen bonding, hydrophobic effects, host–guest recognition, π–π stacking and dynamic coordination bonding, increase effective crosslinking density and compact hydrogel networks. In addition, solvent exchange, tissue fluid absorption and environmental water desorption alter water activity and polymer–water interactions, further triggering hydrogel contraction. Despite diverse contraction mechanisms, all wound-repairing hydrogels translate their material-scale contractile deformation into controllable mechanical forces acting on target tissues [18].

To avoid ambiguity, we define a contractile hydrogel operationally as a hydrogel system in which environmentally triggered network densification generates a measurable, adhesion-transmissible mechanical force capable of drawing wound edges together. This definition deliberately excludes several related but mechanistically and functionally distinct phenomena: (i) passive volume loss caused by uncontrolled dehydration or solvent evaporation, which lacks a defined triggering mechanism and reproducible force output; (ii) simple deswelling of a hydrogel returning from a swollen state to its as-prepared equilibrium volume, which does not involve additional network densification beyond re-establishing equilibrium; (iii) syneresis, i.e., spontaneous expulsion of pore fluid driven by phase separation or aging of the polymer network, which may occur without any external stimulus and is often undesirable in hydrogel storage or processing contexts; and (iv) the natural, cell-driven wound contraction observed in vivo, which is a biological rather than a material process.

Within this scope, we systematically searched the Web of Science Core Collection and PubMed databases for articles published between January 2022 and June 2026, using keyword combinations including “contractile hydrogel,” “shrinking hydrogel,” “stimuli-responsive hydrogel,” “mechanical contraction,” “wound healing,” “skin regeneration,” and “mechanotransduction.” We focus on the fundamental driving forces governing hydrogel contraction and systematically summarize four categories of design strategies, including temperature responsiveness, pH regulation, intermolecular-interaction-induced network densification, and solvent-induced contraction. The underlying mechanisms by which diverse hydrogel systems achieve water expulsion and network contraction through chain collapse, charge rearrangement, dynamic crosslinking reinforcement, and solvent loss are comprehensively elaborated. Furthermore, the biological translation of hydrogel contractile behavior is elucidated at the tissue, cellular, and molecular levels. We further review the applications of contractile hydrogels in the repair of acute full-thickness defects, infected and diabetic chronic wounds, incisional wounds, and irregular dynamic wounds. In addition, the potential and challenges of contractile hydrogels as innovative active mechanical intervention platforms are discussed. This review aims to provide theoretical insights and design guidelines for the further development and clinical translation of high-performance contractile hydrogels for wound healing.

## 2. Design Mechanisms of Contractile Hydrogels

Contractile hydrogels undergo a transition from a loose, water-rich swollen network to a denser network with reduced water content and smaller pores. This transition is accompanied by water expulsion, which in turn gives rise to macroscopic volume or area contraction. Based on the dominant mechanism that initiates this contraction, current systems can be grouped into four categories: temperature-responsive, pH-regulated, intermolecular-interaction-induced, and environmental-medium-induced hydrogels. The representative hydrogel compositions, contraction mechanisms, triggering conditions, and corresponding contraction behaviors are summarized in Table 1. Since multiple stimuli may operate concurrently, we classify by the dominant, rate-limiting physicochemical transition rather than by enumerating all external triggers. For instance, near-infrared (NIR)-responsive poly(N-isopropylacrylamide)(PNIPAM)/GO is grouped as temperature-responsive, as NIR merely delivers heat to raise the gel above its lower critical solution temperature (LCST) while the core mechanism remains dehydration-driven chain collapse. Similarly, solvent-exchange systems are classified as medium-induced when solvent composition or water activity is the primary trigger, with enhanced intermolecular interactions (e.g., hydrogen bonding or hydrophobic aggregation) considered secondary coupled effects. Systems with genuinely co-dominant, independently sufficient driving forces are explicitly treated as hybrid/synergistic (see Section 2.1 and Section 2.4).

### 2.1. Temperature-Induced Contractile Hydrogels

Temperature-responsive contractile hydrogels undergo thermally driven contraction by shifting the balance between polymer–water and polymer–polymer interactions. This behavior is governed by characteristic transition temperatures, typically the lower critical solution temperature (LCST). When the environmental temperature exceeds the LCST, hydrogen bonds between polymer chains and water molecules are weakened or disrupted. Consequently, the polymer chains dehydrate and collapse, the network pores close, both free and bound water are expelled, and the hydrogel deswells with visible macroscopic contraction [39].

**Figure 1:**
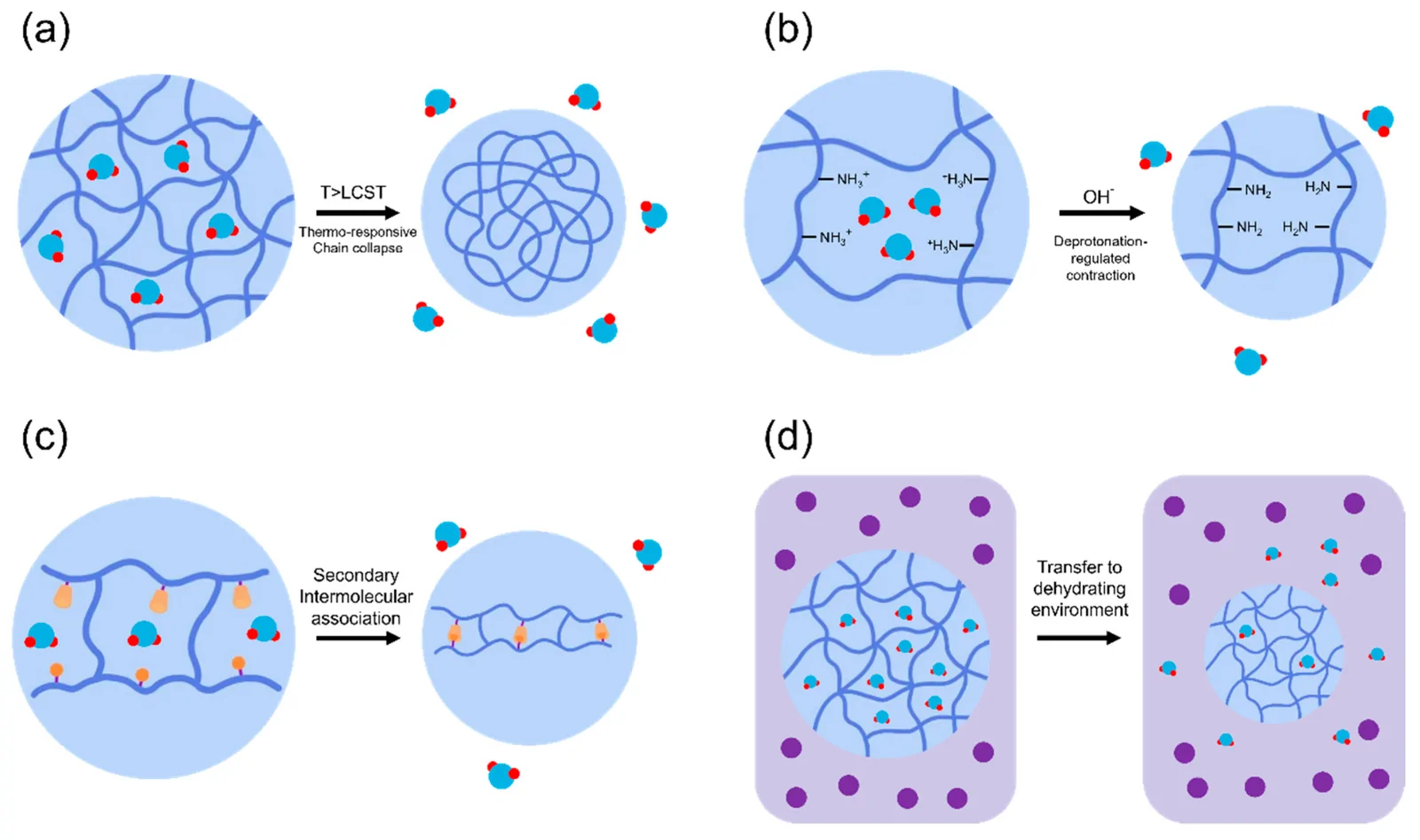
Schematic illustration of the main stimuli-responsive contraction mechanisms in polymer hydrogels. (**a**) Thermoresponsive contraction driven by dehydration and chain collapse above the lower critical solution temperature (LCST). (**b**) pH-responsive contraction resulting from changes in electrostatic repulsion or hydrophobicity upon protonation/deprotonation. (**c**) Contraction induced by specific intermolecular interactions, such as host–guest complexation or hydrogen bonding. (**d**) Contraction triggered by alterations in the solvent quality or environmental medium. Blue curves represent polymer chains, cyan–red clusters represent water molecules, orange symbols represent interacting functional groups or intermolecular association sites, and purple circles represent components of the external dehydrating medium. Changes in the size of the hydrogel domains schematically indicate network contraction and water expulsion.

Among thermoresponsive LCST polymers, poly(N-isopropylacrylamide) (PNIPAM) remains the most widely studied material. Its transition temperature of approximately 32–33 °C lies close to physiological body temperature, making PNIPAM-based hydrogels attractive for wound repair and drug delivery, as they can contract under body-temperature conditions. The mechanism originates from the competition between hydrophilic amide groups and hydrophobic isopropyl groups along the polymer chain (Figure 1a). Below the LCST, amide groups form stable hydrogen bonds with water, keeping the chains hydrated and extended and maintaining a swollen network. Above the LCST, hydrophobic interactions dominate. Polymer–water hydrogen bonding is disrupted, bound water is released, and flexible chains aggregate or entangle, leading to a denser contracted network [40]. However, pure PNIPAM single-network hydrogels are mechanically weak and adhere poorly to wet wound tissue. Therefore, for wound applications, PNIPAM is typically combined with secondary networks or functional components rather than used as an isolated network.

Double-network design offers a key strategy to overcome these limitations. The introduction of a second hydrophilic network can tune mechanical stability while preserving the thermoresponsive contraction of PNIPAM [41]. For instance, Cao et al. constructed a chitosan-modified PNIPAM/sodium alginate double-network hydrogel [42]. This interpenetrating architecture improved structural stability without compromising the intrinsic thermoresponsive contraction of PNIPAM. After incubation at 37 °C for 1 h, the hydrogel retained only about 42–43% of its original area, corresponding to an area contraction strain of approximately 57–58%.

The introduction of functional nanofillers into thermoresponsive contractile hydrogels not only enhances photothermal controllability, but also imparts antibacterial activity and improved adhesion [43,44,45]. For example, graphene oxide and polydopamine can be incorporated into PNIPAM networks as photothermal components. Under near-infrared (NIR) irradiation, these materials rapidly elevate the local gel temperature, reducing reliance on bulk environmental heating. This accelerates chain dehydration and collapse, enabling remotely controlled contraction. Cao et al. developed a PNIPAM/sodium alginate/graphene oxide ternary composite hydrogel, in which sodium alginate formed a reinforcing interpenetrating network and graphene oxide imparted NIR responsiveness [42]. Li et al. further integrated quaternized chitosan and polydopamine-coated reduced graphene oxide into the PNIPAM matrix [24]. This hydrogel combined thermoresponsive contraction, wound adhesion, broad-spectrum antibacterial activity, and photothermal response. After incubation at 37 °C for 60 min, the hydrogel area decreased to 34% of its initial area, demonstrating rapid contraction alongside multiple wound-relevant functions.

Due to the inherent crosslinking mechanism of PNIPAM-based thermoresponsive systems, which typically require prefabrication into patch formats, their conformability to irregular wound geometries is intrinsically limited. This geometrical mismatch not only compromises interfacial contact but may also lead to heterogeneous force transmission and suboptimal wound closure, particularly in clinically complex scenarios involving curved anatomical sites or dynamic wound margins. Consequently, increasing attention has been directed toward thermoresponsive hydrogels with shape-adaptive capabilities and, more ambitiously, injectable in situ–forming hydrogels that can seamlessly conform to wound beds of arbitrary topography [46,47,48,49,50]. For instance, a thermoresponsive hydrogel with shape adaptability has recently been engineered to improve conformal contact with complex wound geometries, demonstrating enhanced wound coverage and superior healing outcomes compared to conventional prefabricated patches [51]. Injectable thermoresponsive hydrogels with combined contractile functionality remain rarely reported in the literature. In contrast, calcium ion-crosslinked alginate hydrogels and pH-responsive chitosan-based systems have been demonstrated to possess both contractile capacity and injectability, providing feasible reference paradigms for related research [35,52,53,54,55]. This research gap underscores the urgent need to develop advanced hydrogel dressings that synergistically integrate contractile performance with injectable delivery.

### 2.2. pH-Regulated Contractile Hydrogels

pH-responsive hydrogels contract primarily through the reversible ionization and deionization of ionizable groups. Changes in environmental pH alter the fixed charge density, electrostatic repulsion, and osmotic pressure within the network, driving a swelling-to-contraction transition. Based on the type of ionizable group, these materials are broadly classified into anionic carboxyl-containing systems and cationic amino-containing systems [56].

Anionic hydrogels are typically constructed from polymers rich in carboxyl groups. Under neutral or alkaline conditions, these groups largely dissociate into negatively charged carboxylate ions. This increases the fixed negative charge density, electrostatic repulsion between chains, and osmotic pressure, drawing water into the network and causing swelling [57,58]. As the pH shifts toward acidic conditions, carboxylate ions become protonated to form neutral carboxyl groups. The loss of fixed charge weakens electrostatic repulsion and reduces the osmotic pressure difference across the gel. Meanwhile, protonated carboxyl groups can form intra- and intermolecular hydrogen bonds, drawing polymer chains closer together. Consequently, the shrinking network pores expel water and drive macroscopic hydrogel contraction [59,60,61].

For example, Liao et al. prepared an HCMC/Fe_3_O_4_ composite hydrogel whose contraction was governed by carboxyl deionization [33]. At pH 1.0, the hydrogel diameter decreased from 1.22 cm to 0.86 cm within 3 h, corresponding to a volume contraction ratio of 29.87%. Li et al. constructed an MA-HA/AA acid-responsive hydrogel that utilized acid-induced contraction to regulate drug release at wound sites [34]. In the acidic microenvironment, carboxyl protonation densified the network, compressed the pores, and expelled pore water; consequently, ginsenoside Rg1 loaded in the pores was released with the outward water flow and diffused to the wound area.

Cationic amino-containing hydrogels exhibit the opposite pH response, contracting under alkaline conditions through deprotonation. chitosan-based hydrogels exhibit high swelling in acidic environments, which is attributed to the protonation of amino groups on chitosan chains into positively charged ammonium groups. As pH rises, ammonium groups are deprotonated back to neutral amino groups. Charge density and chain repulsion diminish, inducing polymer chains to contract via hydrophobic interactions and intermolecular hydrogen bonding [56,62].

Although pH-responsive contraction provides an attractive strategy for programming hydrogel deformation, the physiological relevance of reported systems should be carefully considered. Several pH-responsive contractile hydrogels have been designed to undergo rapid contraction under strongly acidic conditions, which are more representative of gastric environments than cutaneous wounds [34]. These studies provide important insights into pH-triggered network rearrangement, volume transition, and mechanically active deformation; however, their direct application in skin wound management remains limited because the pH range of cutaneous tissues is generally close to neutral or mildly acidic. Therefore, such systems should be regarded primarily as demonstrations of pH-mediated contractile mechanisms, while further optimization toward physiologically compatible pH responsiveness is required for translation into cutaneous wound dressings.

### 2.3. Network Contraction Induced by Intermolecular Interactions

The aforementioned pH- and temperature-responsive hydrogels rely on well-defined external physicochemical stimuli, undergoing volumetric contraction through changes in the ionization state of pendant groups or shifts in the hydrophilic–hydrophobic balance of polymer segments. In contrast, spontaneous network contraction can occur in certain hydrogels under mild or even negligible external stimulation. Unlike their responsive counterparts, this contraction process does not require phase transitions or ionization/dissociation events of specific functional groups. Instead, the primary driving force originates from the dynamic evolution of intermolecular interactions within the gel network. As the mutual interactions among polymer chains progressively strengthen relative to polymer–water interactions, the number of dynamic physical crosslinks within the network increases continuously. This reinforcement constrains chain mobility, gradually reduces the average pore size, and promotes the sustained expulsion of entrapped water from the pores. Consequently, the gel spontaneously transitions from a highly swollen, loose structure to a compact, densified state [63,64]. This contraction behavior is fundamentally attributable to the spontaneous reorganization of the intrinsic network connectivity, and is therefore designated as intermolecular-interaction-induced network densification contraction. A wide variety of intermolecular forces participate in regulating this process, including hydrogen bonding, hydrophobic interactions, π–π stacking, metal coordination, host–guest recognition, and dynamic covalent bonds. Notably, the contraction kinetics and application characteristics differ considerably among these distinct interaction types [64,65,66,67,68].

Hydrogen bonding, hydrophobic interactions, and aromatic stacking represent common driving forces for spontaneous hydrogel contraction [69,70]. Hydrogels bearing hydroxyl, carboxyl, amide, polyphenol, or other polar groups are capable of forming cooperative hydrogen bonds within the network. Polymers or low-molecular-weight gelators that contain aromatic or hydrophobic moieties can further densify the network through hydrophobic aggregation and aromatic stacking [71,72,73]. Duraisamy et al. prepared the small-molecule hydrogelator Fmoc-β-Phe, which contains a fluorenylmethoxycarbonyl aromatic group and a β-phenylalanine structure [65]. After gelation, the hydrogel spontaneously dehydrated and contracted without additional temperature change, pH change, or near-infrared irradiation. This behavior was associated with rearrangement of Fmoc aromatic aggregation from a relatively loose J-type mode into a tighter H-type packing mode. The change in molecular packing reconstructed the loose fibrous scaffold into a denser, more entangled network. Mondal et al. designed three aromatic amino-acid-based tripeptide amphiphilic gelators, G1, G2, and G3 [66]. By changing the position of phenylalanine in the tripeptide chain, they altered the distribution of hydrophobic groups and the efficiency of aromatic stacking. G1 released more than 90% of its pore water within 3 h and contracted rapidly, whereas G2 and G3 released 80–90% of their internal water only after about 7 d. These results indicate that molecular packing, hydrophobic-group distribution, aromatic stacking, and van der Waals interactions strongly determine the rate of nanofiber rearrangement and, consequently, the speed of hydrogel contraction.

Metal ion coordination provides stronger and more localized bonding than many weak interactions and can directly increase effective crosslinking density. Calcium alginate hydrogel is a typical self-contracting system. Alginate is an anionic natural polysaccharide composed of mannuronic acid and guluronic acid units. Calcium ions coordinate specifically with guluronic-acid-rich segments and form egg-box-like physical crosslinking domains [74,75,76]. Da Silva Pinto et al. examined the time-dependent structure of calcium alginate hydrogels and found that highly crosslinked calcium alginate gels can contract spontaneously after gel formation without thermal stimulation [67]. The contraction was attributed to continued accumulation of local coordination domains and secondary rearrangement of polymer chains within the gel.

Host-guest recognition introduces a different type of dynamic control. Its main value is not simply stronger chain association, but reversible and programmable crosslinking through selective recognition between host and guest molecules. Cyclodextrins, pillararenes, and cucurbiturils can include guest molecules such as adamantane, azobenzene, or ferrocene, forming dynamic hydrogel networks [77,78]. Iudin et al. constructed a double-crosslinked hydrogel composed of cyclodextrin-modified methacrylated hyaluronic acid and adamantane-modified methacrylated dextran, denoted HAMA-CD/DexMA-Ad [68]. Methacrylate groups formed a covalent supporting network, while β-cyclodextrin/adamantane interactions acted as reversible crosslinking units. During gelation, adamantane carboxylic acid was introduced as a competitive small-molecule guest. It temporarily occupied cyclodextrin cavities and suppressed host-guest crosslinking between polymer chains. After transfer into phosphate-buffered saline or cell culture medium without the competitive guest, adamantane carboxylic acid diffused out, host-guest binding between HAMA-CD and DexMA-Ad was re-established, effective crosslinking density increased, and polymer chains were pulled inward. With 2 wt% initial polymer content, a CD/Ad molar ratio of 1:1, and 4 mg/mL competitive guest, the hydrogel achieved an approximately eightfold volume contraction and about 50% diameter contraction. In cell-laden tubular hydrogels, the channel diameter decreased from 430 ± 10 μm to 210 ± 10 μm. This example shows how competitive binding and dynamic recognition can convert molecular-level reorganization into controllable macroscopic contraction.

### 2.4. Network Contraction Induced by Solvent Exchange

Solvent- or environmental-medium-induced contraction is governed by changes in external solvent composition, water activity, or the aqueous environment surrounding the gel. In the hydrated state, polymer chains are sufficiently solvated and maintain a swollen network. Upon alteration of the external medium, chain hydration diminishes, polymer–water hydrogen bonding is disrupted, and intrinsic interactions among polymer chains become dominant. Bound and free water are gradually expelled, resulting in macroscopic contraction [79]. Organic-solvent exchange offers a convenient approach for in vitro hydrogel pretreatment, microstructure fixation, or mechanical reinforcement. However, many organic solvents are irritating or cytotoxic and are therefore unsuitable for direct contact with open wounds [80]. In the context of wound repair, physiologically compatible medium-induced contraction holds greater relevance, particularly for systems driven by protein secondary-structure maturation or by dynamic changes in wound humidity and tissue fluid [81].

Physiological-buffer incubation can induce slow and controllable contraction in protein-based hydrogels through secondary-structure ordering [82]. Lee et al. constructed a visible-light-curable silk fibroin hydrogel using riboflavin as the visible-light initiator, with gelation mediated by dityrosine bond formation [38]. After incubation in phosphate-buffered saline at 37 °C, silk fibroin chains underwent conformational transition from random coils toward stable β-sheet structures. These ordered β-sheets promoted close chain packing, compressed the pores, expelled water, and produced overall hydrogel contraction with dimensional deformation of three-dimensional printed structures. Gelatin incorporation suppressed excessive β-sheet formation, reduced packing density, and improved the dimensional stability of printed hydrogels.

Humidity-responsive hydrogel fibers are closer to the wound environment because their contraction follows the cycle of exudate absorption and water evaporation. Dong et al. developed a composite functional fiber composed of sodium alginate, gelatin, and silica nanoparticles [83]. Its response came from reversible hydrogen-bond reorganization. After the fiber absorbed wound exudate, water entered the system and disrupted pre-existing intermolecular hydrogen bonds, allowing polymer chains to extend. As water evaporated and desorbed, hydrogen bonds re-formed, chains retracted, and the fiber contracted axially. The fiber generated a maximum contraction strain of 15% and a maximum isometric contraction stress of 24 MPa. When woven into a wound repair textile, axial contraction of individual fibers was converted into radial contraction of the fabric. In a moist-wound simulation, the fabric absorbed water and then contracted during water evaporation; its radial length decreased from 25.2 mm to 21.5 mm, and the steady contraction force exceeded 1 N. This design illustrates how environmental water dynamics can be converted into mild but sustained mechanical traction.

The advantages of environment-medium-induced contraction are evident. Its triggering mechanism can respond to the natural moisture fluctuations of the wound site [84,85,86]. Moreover, many such systems operate without external energy input, thereby offering favorable physiological compatibility [38,83]. Nevertheless, this strategy faces multiple practical constraints. Some systems require the use of irritant organic solvents, which precludes their direct application to open wounds [79]. Systems relying on natural dehydration are susceptible to ambient humidity, exudate volume, and dressing coverage, leading to inconsistent contraction performance [87]. Additionally, systems based on protein or polysaccharide structural maturation may undergo excessive contraction, which reduces scaffold pore size. Such pore size reduction may restrict cell infiltration and limit the available space for growth, ultimately impairing tissue regeneration [38,88].

The aforementioned contractile hydrogels, each operating through distinct shrinkage mechanisms, exhibit considerable capacity to generate mechanical forces directed toward wound sites. Their functional performance is determined by the underlying contraction mechanisms, kinetics, force generation, and environmental responsiveness. Thermoresponsive systems offer rapid and reversible contraction but require precise temperature regulation, whereas pH-responsive systems provide endogenous responsiveness but are limited by the relatively narrow pH variation of cutaneous wounds. Intermolecular-interaction-driven and solvent-mediated systems enable spontaneous or sustained contraction, although their contraction rate and network stability require further optimization. A comparative summary of the advantages, limitations, contraction behaviors, and potential applications associated with each strategy is presented in Table 2.

## 3. Mechanisms of Contractile Hydrogels for Wound Repair

**Figure 2:**
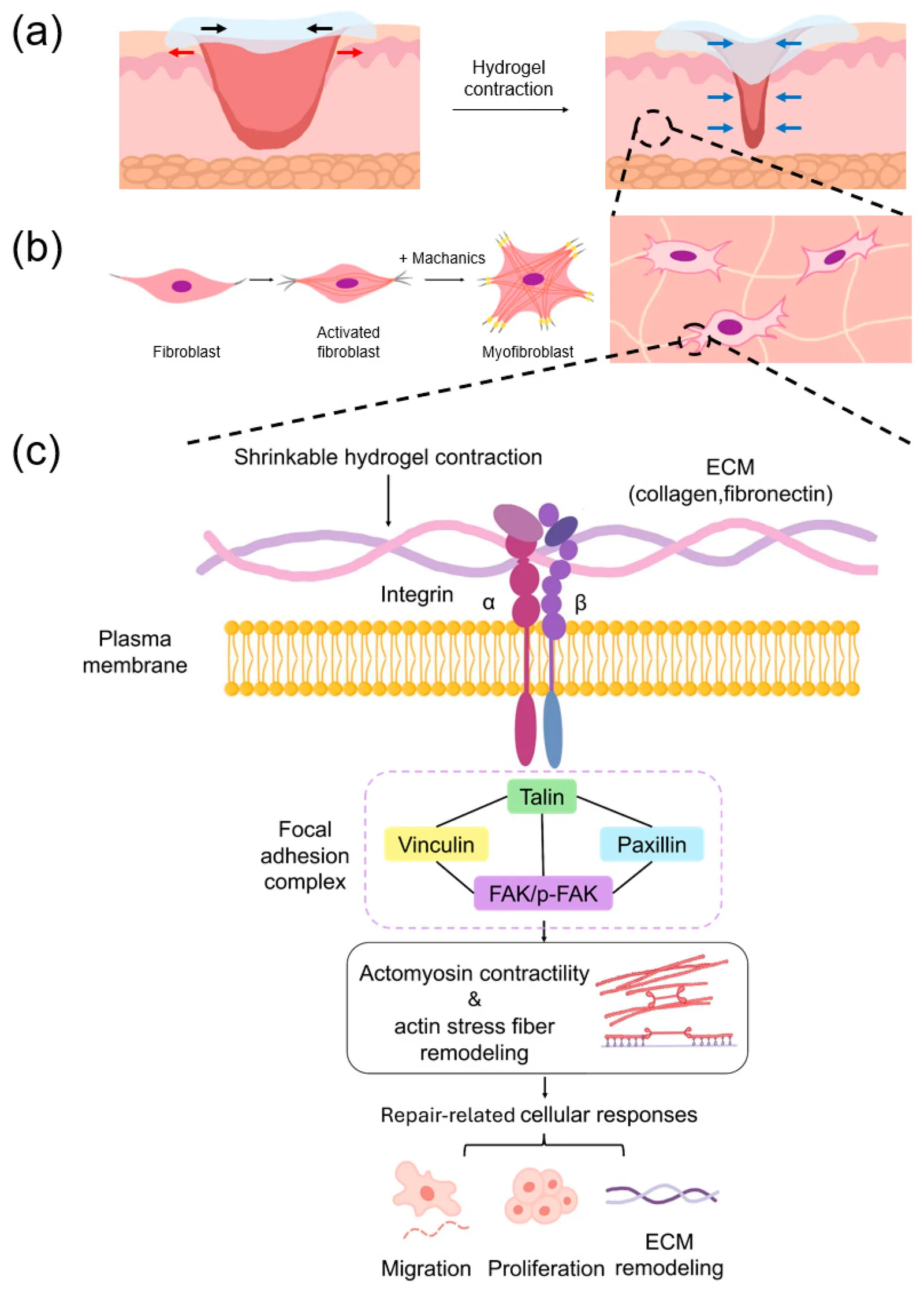
Schematic of mechanotransduction cascade from tissue to molecule. (**a**) Tissue-level contraction reduces wound area via centripetal traction). Red arrows indicate pre-existing outward skin tension, black arrows above the dressing indicate the direction of hydrogel contraction, and blue arrows indicate the resulting centripetal traction on the wound edges. (**b**) Cellular-level mechanical signals regulate fibroblast cytoskeletal dynamics and matrix remodeling. (**c**) Molecular-level mechanosensing involves integrin-mediated focal-adhesion signaling, linking ECM-derived mechanical cues to actomyosin contractility, actin stress-fiber remodeling, and repair-related cellular responses. Black dashed lines denote magnified regions, whereas solid arrows indicate phenotypic progression or the direction of mechanotransduction.

Hydrogel contraction becomes biologically effective only when material deformation is converted into mechanical force and transmitted across a stable hydrogel–tissue interface. The resulting tissue and extracellular matrix deformation can alter wound geometry, local stress distribution, and the mechanical microenvironment of resident cells (Figure 2). At the tissue level, effective interfacial coupling enables contractile hydrogels to generate wound-edge traction and redistribute local mechanical loads. At the cellular level, these transmitted forces can influence cell shape, membrane tension, focal adhesion organization, and cytoskeletal tension, thereby regulating repair-related cellular responses. At the molecular level, current evidence most consistently supports the involvement of focal-adhesion-associated signaling in selected mechanically active wound systems, whereas other mechanosensitive pathways are supported mainly by broader mechanobiological studies. This section therefore examines the effects of contractile hydrogels across tissue, cellular, and molecular scales, with particular attention to the strength and experimental context of the available mechanistic evidence.

### 3.1. Tissue Level: Wound Geometry Closure and Local Tension Remodeling

At the tissue scale, contractile hydrogels can promote wound-edge approximation when contraction-generated force is effectively transmitted through the hydrogel–tissue interface (Figure 2a). Geometric closure shortens the distance that keratinocytes must migrate toward the wound center and reduces the amount of granulation tissue and extracellular matrix required to fill the defect. When conformal contact and sufficient adhesion are achieved, contraction may also reduce superficial gaps, fluid accumulation, and interface instability [28,89]. For example, Chen et al. developed a PAA/M-HPMC skin stress-shielding hydrogel that converts contraction into wound mechanical microenvironment regulation [27]. The dressing integrates thermoresponsive M-HPMC as the contractile unit and a PAA network for wet-tissue adhesion. Upon contact with periwound skin, body temperature triggers hydrogel contraction, enabling edge gripping and stress redistribution. The material achieved 31.2% area shrinkage at 37 °C over 5 h, with ~8.3 kPa shear adhesion to porcine skin that remained ~7.5 kPa after five cycles, confirming its capacity for contraction–traction coupling. In full-thickness defects, the dressing reduced periwound stress concentration beyond physical coverage, suppressed fibroblast mechanoactivation, and downregulated TGF-β1, collagen I, and integrin/FAK/p-FAK signaling, thus limiting ECM overdeposition and scar formation.

The efficiency, orientation, and duration of force transmission are the primary determinants of tissue-level outcomes for contractile dressings. Uniform and moderate traction facilitates precise wound-edge approximation, eliminates dead space, reduces fluid accumulation, and lowers infection risk, while also providing sustained dynamic fixation after closure. Conversely, excessive traction, misaligned force vectors, or inadequate interfacial adhesion may result in ineffective shrinkage, where the hydrogel contracts without displacing wound margins and even damages to nascent granulation tissue. Accordingly, the performance of contractile dressings at the tissue level should not be assessed by the material’s free shrinkage ratio alone, but rather by the magnitude, direction, and duration of the force actually delivered to the wound bed [23,28,90].

### 3.2. Cellular Level: Fibroblast-Centered Multicellular Repair Driven by Mechanical Responses

Changes in wound geometry and local mechanics alter the spatial environment, extracellular matrix stiffness, and mechanical load experienced by reparative cells. These cues regulate proliferation, migration, differentiation, and phenotypic transition. Fibroblasts are especially important because they synthesize, degrade, and remodel extracellular matrix and are highly sensitive to mechanical input. After injury, inflammatory signals, matrix disruption, and mechanical imbalance jointly activate quiescent fibroblasts, increasing their migration, proliferation, collagen secretion, and contractile capacity [91,92]. Some fibroblasts transition into myofibroblasts, a process supported by actomyosin stress fibers and associated with wound contraction and matrix remodeling (Figure 2b). Transient and moderate myofibroblast activation contributes to repair, whereas persistent activation drives excessive extracellular matrix deposition and pathological scarring [17].

Early moderate centripetal traction reduces the wound defect cavity and diminishes the volume of granulation tissue required for fibroblast-mediated repair. Concurrently, this mechanical stimulus alters the tensile environment of the extracellular matrix, promoting fibroblast adhesion, spreading, directional migration, and ordered matrix deposition [93,94]. As illustrated in Figure 2b, mechanical contraction drives fibroblasts from a quiescent state characterized by low tension and weak contractility toward an activated phenotype with enhanced spreading and contractile activity, accompanied by stress fiber reorganization and augmented actomyosin contractility, thereby facilitating granulation tissue maturation and wound contraction. This mechanomodulation, however, operates within a defined temporal window. Prolonged or excessive traction sustains a high-stiffness, high-stress extracellular matrix microenvironment, leading to persistent accumulation of α-SMA-positive myofibroblasts and upregulated collagen I expression, which collectively impose a significantly elevated risk of hypertrophic scar formation [95].

Li et al. developed a GNA thermoresponsive adhesive mechanoactive hydrogel composed of GelMA, PNIPAM, and AA. In this system, PNIPAM confers temperature-responsive contractility, GelMA provides sites for cell adhesion and matrix remodeling, and PAA enhances tissue adhesion, enabling effective transmission of contractile forces to surrounding cells [96]. Upon contraction at 35 °C, GNA exerted traction on periwound tissues. In a rat full-thickness defect model, GNA significantly accelerated wound closure, promoted epidermal regeneration, and attenuated inflammatory responses. Further mechanistic analysis revealed that GNA increased the proportion of proliferating basal cells, as evidenced by elevated EdU and p63 positivity, while delaying cell differentiation and activating mechanosensitive signaling pathways including MAPK, Wnt, and Hippo. Inhibition of YAP or MEK markedly attenuated the pro-healing effects of GNA, indicating that this contractile hydrogel promotes repair not merely through physical traction, but also via YAP- and MEK/ERK-mediated regulation of cellular behavior to enhance tissue regeneration.

Keratinocytes respond mainly to the reduced wound area and improved interface stability produced by contraction [97]. In large acute wounds and diabetic chronic wounds, delayed re-epithelialization and weak intrinsic skin contraction contribute to prolonged non-healing [98,99]. By reducing the open area, contractile hydrogels shorten the migration distance for keratinocytes and maintain a continuous, moist, and mechanically stable surface for re-epithelialization [100]. Sun et al. constructed a composite material combining amino- and hydroxyl-modified C70 fullerene antioxidants with a thermoresponsive PNIPAM/Alg contractile hydrogel [22]. At 37 °C, the material contracted and thus reduced the open wound area. The contraction-generated mechanical stimulus activated mechanosensitive EGFR/Akt signaling and promoted keratinocyte proliferation. In vivo, the material increased re-epithelialization efficiency by approximately 2.7-fold in acute wounds and 1.7-fold in diabetic chronic wounds.

### 3.3. Molecular Level: Contraction-Driven Mechanosensing, Cytoskeletal Remodeling, and Nuclear Transcriptional Regulation

When contraction-generated force is effectively transmitted across the hydrogel–tissue interface, extracellular matrix and cellular deformation can alter membrane tension, focal adhesion organization, and cytoskeletal tension, thereby initiating mechanosensitive biochemical responses [101,102]. Changes in integrin/FAK-associated signaling have been demonstrated in selected contractile-hydrogel and mechanically active wound-repair systems, whereas the involvement of Piezo1, TRPV4, and related Ca^2+^-dependent signaling is derived mainly from broader wound and cell mechanobiology. Accordingly, focal-adhesion signaling and membrane mechanosensing represent distinct mechanistic branches with different levels of experimental support rather than obligatory sequential pathways converging on a common downstream regulator (Figure 2c).

The integrin/focal adhesion kinase (FAK) pathway serves as a primary mechanotransduction axis through which cells sense matrix tension and external traction [103]. Upon contraction-derived force reaching the cell surface, integrins engage extracellular matrix ligands to initiate focal adhesion assembly [104]. On the intracellular side, structural proteins such as talin, vinculin, and FAK are recruited, facilitating the formation of mature and functionally active focal adhesions [105]. Remodeling of these adhesions modulates FAK phosphorylation and couples mechanical stimuli to downstream signaling cascades, including RhoA/ROCK, PI3K/Akt, and cytoskeletal regulators [101,106]. These pathways influence actomyosin organization, cellular contractility, migration, and matrix remodeling, but they should be viewed as related downstream branches rather than a fully resolved sequence in contractile-hydrogel wound repair.

YAP and TAZ are mechanosensitive transcriptional regulators whose nuclear localization is controlled by cytoskeletal tension, focal adhesion organization, and nuclear mechanics [107]. During early wound repair, moderate contraction cues may promote limited YAP/TAZ nuclear translocation, supporting genes associated with reparative cell proliferation, directional migration, and extracellular matrix remodeling. This can accelerate re-epithelialization and granulation tissue maturation [107,108]. However, under sustained or excessively high traction, pronounced YAP/TAZ nuclear retention can sustain fibrotic gene expression programs, drive aberrant fibroblast activation, and ultimately contribute to pathological scar formation [106,108].

Piezo1 and TRPV4 provide potential routes for membrane-level mechanosensing. Both channels respond to mechanical deformation in defined experimental contexts, but the supporting evidence originates from distinct cell types and loading conditions. Direct measurements in chondrocytes have shown that Piezo1 and TRPV4 respond through different mechanotransduction mechanisms under mechanical loading [102]. Piezo1 has also been implicated in the spatial regulation of keratinocyte migration during wound healing and in the adaptation of vascular architecture to physiological force [109]. These studies support the biological plausibility that contraction-induced changes in tissue deformation, membrane tension, or cell spreading could engage Piezo1- or TRPV4-mediated Ca^2+^ influx in wound-associated cells. The resulting Ca^2+^-dependent signals may influence cytoskeletal remodeling, migration, inflammatory activity, and vascular responses [109].

Mechanical cues also intersect with inflammatory and fibrotic molecular programs. Moderate wound closure can reduce external inflammatory stimulation and support a more orderly transition through the repair stages. Persistent high tension, by contrast, upregulates fibrotic markers and signaling molecules such as TGF-β1, α-SMA, collagen type I, collagen type III, and phosphorylated focal adhesion kinase. TGF-β1 is a central profibrotic regulator that drives fibroblast-to-myofibroblast differentiation and abnormal matrix deposition [110]. By reshaping the wound mechanical microenvironment, dynamic contractile dressings can downregulate TGF-β1, collagen type I, and phosphorylated focal adhesion kinase [106,111]. They may also interact with EGFR/Akt-mediated proliferative signaling and antioxidant biochemical signals, thereby coordinating re-epithelialization, closure, and scar-reducing repair [112].

Beyond its independent regulation of cellular behaviors, mechanical signals generated by contractile hydrogels may interact with biochemical and antioxidant pathways to modulate chronic wound healing outcomes. Mechanical deformation and stress transmission can activate mechanosensitive pathways, such as YAP/TAZ, integrin-mediated focal adhesion signaling, and cytoskeletal remodeling, which subsequently influence inflammatory responses, reactive oxygen species (ROS) metabolism, and ECM remodeling [8,113]. In chronic wounds characterized by persistent inflammation and oxidative stress, appropriate mechanical stimulation may promote macrophage phenotype transition, enhance antioxidant defense, and restore a regenerative microenvironment [114]. Therefore, the therapeutic effects of contractile hydrogels may be considered as an integrated process involving both physical force regulation and biochemical signal modulation.

## 4. Applications of Contractile Hydrogels in Skin Wound Repair

Conventional passive hydrogel dressings primarily function by maintaining a moist wound microenvironment, absorbing wound exudate, and providing a protective physical barrier. However, because they generally lack active mechanical actuation, their ability to directly counteract wound-edge separation and regulate wound geometry remains limited. In contrast, contractile hydrogels introduce an additional mechanical function by generating controlled contraction forces through programmable network reorganization, offering a potential strategy to actively modulate wound morphology and facilitate wound-edge approximation. Through rational material design, contractile hydrogels can further incorporate complementary functionalities, such as antibacterial, anti-inflammatory, and antioxidant properties, together with improved interfacial interactions, expanding their potential applications in diverse wound scenarios, including acute full-thickness skin defects, infected or diabetic chronic wounds, surgical incisions, and scar management (Table 3).

### 4.1. Active Centripetal Traction for Acute Full-Thickness Skin Defect Repair

Acute full-thickness skin defects involve the loss of both epidermis and dermis. The release of intrinsic skin tension causes wound-edge retraction and enlargement of the open area. As a result, keratinocytes must migrate over a longer distance to complete re-epithelialization, while fibroblasts and newly deposited extracellular matrix must fill a larger defect. Contractile hydrogels can reduce the wound area through centripetal traction, thereby shortening the keratinocyte migration path and lessening the burden of granulation tissue formation and matrix deposition [122].

For example, Zhao et al. designed a thermoresponsive adhesive hyaluronic-acid-based hydrogel (PNI-HA) for active mechanical closure of acute full-thickness skin defects [21]. PNIPAM served as the body-temperature-responsive contraction unit, driving the transition from a swollen to a contracted state at 37 °C. A glutaraldehyde-crosslinked hyaluronic acid network was introduced to improve wet adhesion via Schiff-base reactions between aldehyde groups and amino groups on the tissue surface. This adhesive network enabled the conversion of hydrogel contraction into centripetal traction on wound edges. In a mouse full-thickness skin defect model, PNI-1.0HA achieved a wound closure rate of approximately 45.9% within 4 h, outperforming both the single PNI hydrogel and the blank control. Over the 21-day repair process, the PNI-1.0HA group showed about 43.7% wound closure by day 3, along with reduced inflammatory infiltration, increased CD31-positive angiogenesis, and lower collagen deposition on Masson staining. The key design insight is that thermoresponsive contraction becomes more relevant to wound repair when paired with tissue adhesion capable of transmitting force to the wound edge.

**Figure 3:**
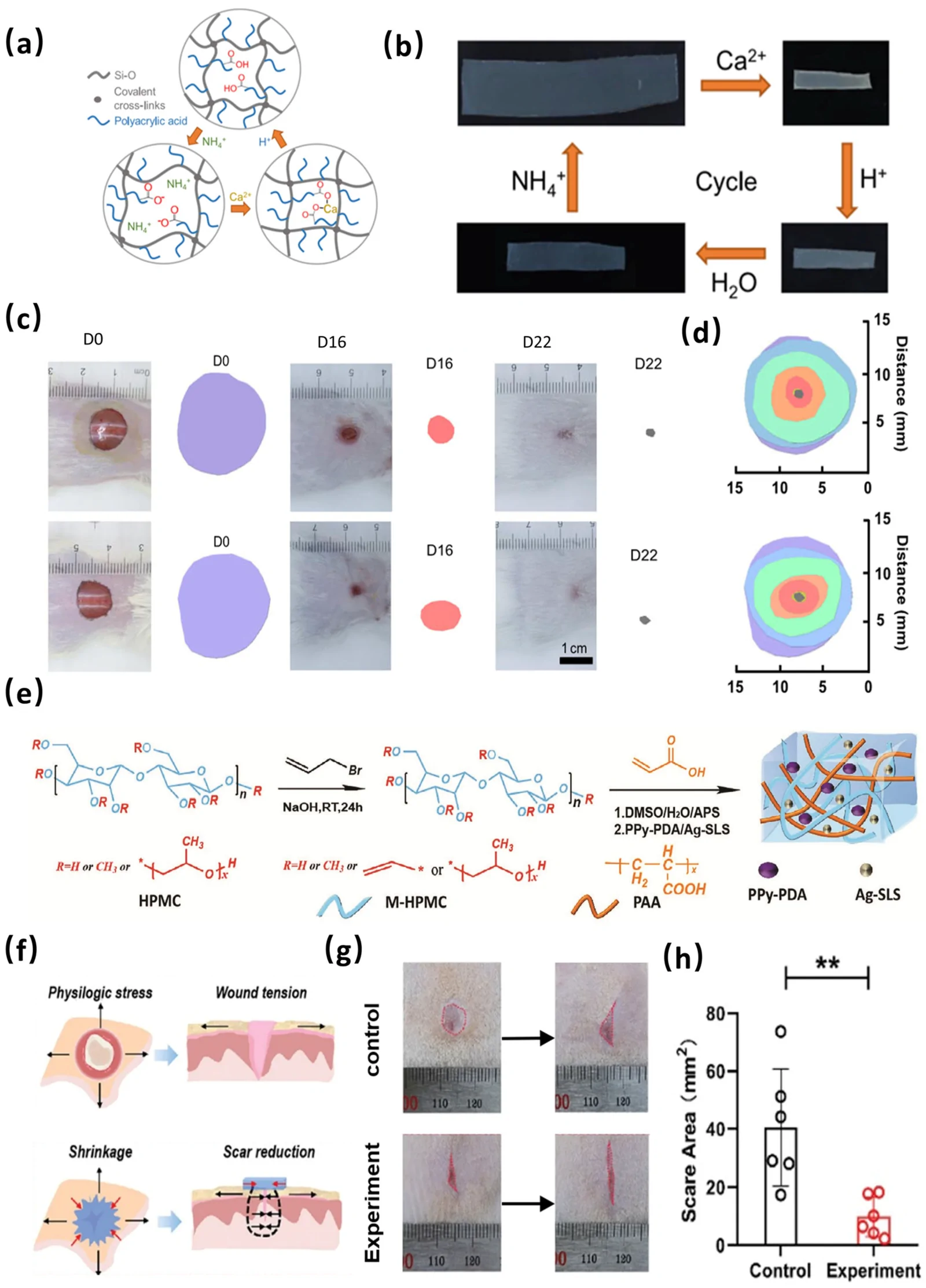
Contractile hydrogel-mediated wound reduction. (**a**) Schematic illustration of the dynamic ionic cross-linking mechanism responsible for the reversible contraction behavior of ion-responsive hydrogels. (**b**) Representative images showing reversible swelling–shrinking cycles of the hydrogel triggered by sequential ionic stimulation. (**c**) Representative photographs and corresponding wound area visualization during the healing process at different time points (D0, D16, and D22). (**d**) Quantitative mapping of wound closure profiles during the regeneration process. (**e**) Schematic illustration of the synthesis process of the shrinking hydrogel dressing based on modified hydroxypropyl methylcellulose (M-HPMC) and polyacrylic acid (PAA) networks. (**f**) Schematic illustration demonstrating that active wound shrinkage enabled by the stress-shielding hydrogel remodels the mechanical microenvironment of wound sites and reduces scar formation. (**g**) Representative photographs of wounds in control and experimental groups before and after treatment with shrinking hydrogel dressings. (**h**) Statistical analysis of scar area in control and experimental groups. Panels (**a**–**d**) were reproduced from Tian et al. [123] under the terms of the Creative Commons Attribution License, whereas panels (**e**–**h**) were adapted from Chen et al. [27] under the terms of the Creative Commons Attribution License. In panel (**a**), gray wavy lines, gray dots, and blue curves represent the Si–O network, covalent cross-links, and poly(acrylic acid) chains, respectively; the colored molecular symbols indicate carboxyl/carboxylate groups and the H^+^, NH_4_^+^, and Ca^2+^ ions shown in the schematic. Orange arrows indicate reversible transitions between different ionic states. In panel (**b**), orange arrows indicate the sequence of exposure to Ca^2+^, H^+^, H_2_O, and NH_4_^+^ environments. Colored wound-area masks in panel (**c**) and nested colored traces in panel (**d**) represent the wound boundaries at successive observation time points. In panel (**e**), blue and orange polymer chains represent M-HPMC and PAA, respectively, whereas purple and gold particles represent PPy-PDA and Ag-SLS; red structural fragments indicate the variable substituent groups of HPMC/M-HPMC, and red asterisks denote their attachment points to the polymer backbone rather than statistical significance. Black reaction arrows indicate the HPMC modification and subsequent polymerization/network-formation steps. In panel (**f**), black arrows indicate outward physiological or wound tension, red arrows indicate inward hydrogel-generated contraction, light-blue arrows indicate the illustrated transition between mechanical states, and the black dashed outline highlights the wound/scar region. In panel (**g**), red dashed outlines delineate the scar boundaries; the photographs are representative day-42 samples from the control and experimental groups and do not represent temporal progression. In panel (**h**), black and red open circles represent individual measurements in the control and experimental groups, respectively; bars and error bars represent the mean ± SD (n = 6 per group), and ** indicates *p* ≤ 0.01.

Despite the benefits of isotropic contraction, it inherently distributes force in all directions rather than concentrating it along the desired wound-closure axis, leading to mechanical loss and reduced efficiency. Anisotropic structural design therefore becomes important when directional contraction is needed. Tian et al. developed a connective-tissue-inspired elastomer-based hydrogel (CEBH) by grafting polyacrylic acid chains throughout a silicone-rubber network [123]. Deprotonation of the carboxyl groups expanded the hydrogel in a weak alkaline solution, whereas Ca^2+^ coordination with carboxylate groups increased the ionic crosslinking density and induced macroscopic dimensional contraction within 60 s. Subsequent exposure to weak acid, water, and NH_4_^+^ enabled reversible shape recovery and reswelling (Figure 3a,b). In a 10 mm full-thickness mouse wound model, CEBH and Tegaderm produced comparable wound-closure trajectories through day 22, and CEBH did not increase inflammatory-cell infiltration or fibrosis (Figure 3c,d). This system therefore demonstrates ion-controlled conformal fixation and reversible mechanical adaptation rather than enhanced wound-closure efficacy.

### 4.2. Synergistic Treatment of Chronic Wounds

Unlike acute wounds, infected and diabetic chronic wounds are characterized by multiple concurrent healing obstacles, including prolonged wound exposure, extensive bacterial colonization, excessive reactive oxygen species (ROS) accumulation, sustained inflammatory activation, impaired angiogenesis, and compromised cell proliferation and migration [124]. In such a complex pathological microenvironment, mechanical closure alone is insufficient to reverse the chronic healing deficit. Therefore, an effective therapeutic strategy for chronic wounds should integrate two complementary functions. Hydrogel network contraction should provide physical wound closure by reducing the exposed wound area, eliminating dead space, and limiting exudate accumulation and bacterial dissemination. Furthermore, additional functional modules, such as antibacterial, antioxidant, and pro-angiogenic components, should be incorporated to address the intrinsic pathological defects of chronic wounds, thereby achieving synergistic mechanical and biochemical repair.

Du et al. developed a multifunctional composite hydrogel (HA/Gel/PNIPAM@IB-367/Ten-2) that integrates mechanical closure, antibacterial activity, and pro-angiogenic function [117]. The hyaluronic acid/gelatin dynamic network provided a moist and conformable wound interface, the PNIPAM thermoresponsive component generated body-temperature-triggered contraction forces, the antimicrobial peptide IB-367 efficiently eliminated methicillin-resistant Staphylococcus aureus (MRSA), and the Ten-2 protein specifically ameliorated angiogenesis deficits in diabetic wounds. In vivo experiments demonstrated that by postoperative day 7, the wound area in the PNIPAM-containing contraction group was 0.13 ± 0.004 mm^2^, substantially smaller than that in the non-contractile control group (0.22 ± 0.004 mm^2^). Furthermore, with the incorporation of antibacterial and pro-angiogenic modules, the wound closure rate reached 91% by day 14, significantly outperforming the blank control. In addition, the hydrogel upregulated the expression of angiogenesis markers (CD31, VEGF-A), collagen deposition marker (Collagen I), cell proliferation marker (Ki67), and M2-type anti-inflammatory macrophage marker (CD206).

Oxidative stress is another major barrier in diabetic wound repair. Excess reactive oxygen species inhibit reparative cell proliferation, induce apoptosis, and aggravate inflammation [22,124]. Therefore, when adapting contractile hydrogels for chronic wounds, incorporating antioxidant functionality becomes essential. Sun et al. combined a thermoresponsive actively contractile hydrogel with amino- and hydroxyl-modified fullerene antioxidants [22]. The thermoresponsive gel contracted efficiently at body temperature, whereas the modified fullerene scavenged excessive reactive oxygen species and relieved oxidative stress. The composite gel showed a volume contraction ratio of 73.70% within 1 h at 37 °C. In an acute wound model, the contractile gel alone increased the healing rate by 13% compared with the blank group. In refractory diabetic wounds, the high-dose composite gel group reached 60% closure on postoperative day 9, whereas the blank control reached only 23%. The dressing increased CD206 expression by 8.4-fold and CD31 expression by up to 6.1-fold, and it improved the ordered deposition of collagen types I and III.

Contractile dressings are increasingly being integrated with diagnostic functionalities. Zhong et al. developed an intelligent NGPR-Zn hydrogel that combines body-temperature-responsive contraction, antibacterial and antioxidant activities, and real-time wound pH monitoring [115]. In the NGPR-Zn hydrogel, PNIPAM provided temperature-controlled contraction, zinc ions contributed broad-spectrum antibacterial and pro-repair effects, and red cabbage extract served as a natural pH-responsive colorimetric indicator. In a bacteria-infected acute wound model, the residual wound areas in the dressing group were 66.46%, 45.37%, and 13.13% on postoperative days 3, 7, and 10, respectively—consistently outperforming the zinc-free control group. The dressing also promoted ordered collagen deposition. In diabetic wound repair, the hydrogel facilitated the reconstruction of tissue architecture resembling that of normal skin, along with hair follicle regeneration and well-aligned collagen fiber arrangement. Notably, RGB color values obtained after 30 min of wound contact showed strong concordance with commercial pH test strips, enabling noninvasive real-time pH tracking. Collectively, these studies suggest that for infected and diabetic chronic wounds, the efficiency of contractile hydrogels lies in the synergistic combination of mechanical closure and modular microenvironmental regulation.

### 4.3. Incision Repair and Scar Control

Skin incisions differ mechanically from large open full-thickness defects. They are continuously pulled by surrounding normal skin and prone to stress concentration perpendicular to Langer lines [125]. Persistent abnormal tension activates fibroblasts and promotes their transition into myofibroblasts, leading to excessive and disordered extracellular matrix deposition and hypertrophic scarring [125,126]. Contractile hydrogels for incision repair and scar control can generate directional contraction to offset outward skin tension and create a lower-stress healing environment. Chen et al. designed the stress-shielding thermoresponsive PAA/M-HPMC hydrogel for scar-reducing repair in high-tension skin wounds (Figure 3e) [27]. Modified hydroxypropyl methylcellulose provided body-temperature-responsive contraction, while the polyacrylic acid network and surface carboxyl groups improved wet adhesion and stable force transmission to wound edges (Figure 3e,f). In a 10 mm full-thickness wound model in SD rats, the experimental group exhibited a markedly smaller residual scar than the control group after 6 weeks (Figure 3g,h).

For linear incisions, the direction of mechanical output is especially important. Conventional thermoresponsive hydrogels usually contract isotropically and cannot precisely compensate for uniaxial outward tension. Ma et al. addressed this limitation by preparing an anisotropic PNIPAM/sodium alginate contractile network through stretch fixation, combined with a dynamic Schiff-base inner gel loaded with photothermal nanozymes [29]. The resulting bilayer directional contractile hydrogel reduced abnormal stress around the incision by 90.3% and generated a directional contraction stress of 27.6 kPa. In a 1.5 cm linear skin incision model, complete incision healing was achieved by postoperative day 10, with almost no visible surface scar. These results highlight the advantage of anisotropic design in guiding contraction forces along the desired axis, thereby promoting scarless incision healing.

Notably, the interpretation of wound closure outcomes from rodent models requires caution. Due to the presence of the panniculus carnosus muscle, mice and rats exhibit substantial wound contraction that differs from human skin repair, which relies more on granulation tissue formation and re-epithelialization. Therefore, splinted wound models and large-animal models, such as porcine models with closer skin biomechanical properties to humans, are valuable for more accurately evaluating the clinical potential of contractile hydrogels. Additionally, mechanical wound closure should not be directly equated with functional tissue regeneration. A reduction in visible wound area primarily represents macroscopic contraction or edge approximation and does not necessarily indicate restoration of tissue architecture and function. Although rapid contraction may contribute to early wound-size reduction, comprehensive evaluation of contractile hydrogels should also consider regenerative outcomes, including re-epithelialization, restoration of skin appendages, vascular maturation, collagen remodeling, mechanical strength recovery, and scar quality. Thus, the development of contractile hydrogels requires not only optimization of contraction behavior but also integration of mechanical regulation with biological regeneration.

## 5. Summary and Perspectives

This review summarized the design principles and contraction mechanisms of four categories of shrinkable hydrogels, including temperature-responsive, pH-regulated, intermolecular-interaction-mediated, and solvent- or environmental-medium-induced systems. Across all these systems, the core physicochemical event remains the transition from a swollen, water-rich network to a densified state characterized by reduced pore size and water expulsion. The biological efficacy of these materials relies on effective conversion of network densification into wound-edge traction through interfacial adhesion. Mechanistically, contraction-derived cues are transduced via integrin/FAK-mediated focal adhesion and Piezo1/TRPV4-mediated membrane mechanosensing, regulating cell proliferation, migration, inflammation, angiogenesis, and fibrosis across tissue, cellular, and molecular scales. Therapeutic applications span acute defects, infected and diabetic chronic wounds, surgical incisions, and scar prevention.

Although hydrogel contraction provides the fundamental driving force for mechanical regulation, the therapeutic relevance of this behavior depends on whether the generated deformation can be effectively converted into tissue-level traction. Therefore, contraction performance should not be evaluated solely by free shrinkage magnitude [127]. Effective wound closure requires stable wet-tissue adhesion to anchor the hydrogel and minimize interfacial slipping, as well as efficient force transmission across the hydrogel–tissue interface. In addition, the spatial orientation of contraction forces is critical, because uncontrolled isotropic shrinkage may result in material deformation without productive wound-edge approximation. The activation time and contraction kinetics should also be carefully balanced: excessively rapid contraction may compromise interface stability or induce excessive mechanical stress, whereas insufficient contraction speed may fail to provide early mechanical support. Furthermore, maintaining contractile function under physiological conditions remains challenging, as continuous exposure to wound exudate, proteins, enzymes, and ions may alter swelling behavior, network integrity, adhesion strength, and stress relaxation, ultimately leading to attenuation of effective traction.

Furthermore, contractile hydrogels present considerable challenges in biosafety, particularly for clinical translation. Sustained contractile stress may compress peri-wound capillaries below the perfusion threshold, leading to localized ischemia and delayed granulation, particularly in diabetic or vascularly compromised patients, while the strong interfacial adhesion required for efficient force transmission simultaneously raises the risk of tissue tearing at the wound margin during removal or unintended shear, creating an inherent trade-off between adhesion strength and atraumatic detachment that current designs rarely address. In addition, energy-triggered systems, such as near-infrared-responsive hydrogels, carry a potential risk of localized thermal injury. Moreover, degradation of the contracted network may release oligomeric byproducts. The long-term biocompatibility of these degradation products remains largely uncharacterized. These concerns suggest that future biosafety evaluations should not rely solely on conventional cytotoxicity assays. Instead, they should incorporate mechanically explicit endpoints, including perfusion monitoring of peri-wound tissues, interfacial fracture testing, and analysis of dose–injury relationships [128,129,130].

Beyond biological efficacy, the clinical translation of contractile hydrogels requires addressing several practical challenges. Large-scale manufacturing should ensure reproducible network structures, contraction performance, and batch-to-batch consistency. Sterilization methods must preserve hydrogel integrity and mechanical activity, while long-term storage stability remains critical for maintaining functional performance. In addition, regulatory approval requires comprehensive evaluation of material safety, degradation behavior, and mechanical effects under clinically relevant conditions. Finally, production cost, manufacturing complexity, and scalability will strongly influence the commercial feasibility of contractile hydrogel-based wound dressings.

Future research should move beyond the unidimensional goal of maximizing shrinkage magnitude and instead pursue the integrated optimization of contraction behavior, tissue adhesion, force transmission efficiency, directional traction, and long-term mechanical stability. Concurrently, several translational barriers must be addressed, including developing injectable hydrogels with intrinsically contractile networks, optimizing reversible adhesion performance, and rigorously evaluating the long-term biosafety of functional nanomaterials, all of which are prerequisites for clinical adoption. Emerging fabrication and computational approaches offer transformative potential in realizing these goals. Programmable hydrogel systems, for instance, enable precise spatiotemporal control over contraction magnitude, activation kinetics, and deformation patterns, thereby affording sophisticated manipulation of wound geometry. In parallel, four-dimensional (4D) printing techniques permit the construction of spatially patterned constructs with predetermined shape-morphing behaviors and dynamic mechanical profiles [131]. Artificial-intelligence-driven design, coupled with data-driven modeling, can further accelerate optimization by establishing predictive correlations among compositional parameters, network architectures, mechanical properties, and biological outcomes [132,133]. Looking forward, contractile hydrogel platforms are poised to evolve toward self-regulating or feedback-responsive systems capable of adapting their mechanical outputs to dynamic changes in the wound microenvironment. Integration with wearable biosensing technologies could enable real-time wound assessment and personalized therapeutic modulation, ultimately fostering the development of intelligent, patient-tailored wound care strategies [134]. Collectively, contractile hydrogels represent a paradigm shift from passive wound coverage to active mechanical intervention. Through synergistic advances in material design, mechanistic dissection, physiologically relevant validation, and smart technology integration, this class of biomaterials holds promise to fundamentally transform the clinical management of chronic and complex wounds by harnessing the power of mechanical forces.

## Figures and Tables

**Table 1:** Classification of representative contractile hydrogels and their stimuli-responsive behaviors.

Hydrogel System	Responsive Mechanism	Stimulus	Reported Contraction Parameter	Contraction Ratio	Reversibility	Refs
PNIPAM	LCST-induced contraction	32 °C (<37 °C)	Volume contraction	64.2% (8 h)	Yes	[19]
PNIPAM/GelMA		37 °C	Area contraction	52.8–56.5% (1 h)	Yes	[20]
PNIPAM-HA		33 °C (<37 °C)	Area contraction	26.6% (2 h); 45.9% (4 h)	Yes	[21]
PNIPAM/Alg/ MBAA/CaCl_2_		37 °C	Volume contraction	73.7% (1 h)	Yes	[22]
PNIPAM/THMA/ GelMA/ ε-PL/GA		37 °C outer layer; 25 °C inner layer	Area contraction	43.3% (1 h)	Yes	[23]
QCS/rGO-PDA/PNIPAM		37 °C	Area contraction	56.5%(2 h)	Yes	[24]
QCS/PNIPAM/PAA/ PAMPS		37 °C	Area contraction	21% (1 h); max. 43.1% (6 h)	Yes	[25]
PVA/TA/PVA-MA-g-PNIPAM		30 °C (<37 °C)	Mass contraction	13.3 → 2 (12 h)	Yes	[26]
M-HPMC/PAA/PPy-PDA		37 °C	Area contraction	31.2% (5 h)	Yes	[27]
PNIPAM/alginate/ Ag-NPs/CaSO4		37 °C	Area contraction	64% (3 h)	Yes	[28]
DCTH/PH	Photothermal- induced contraction	808 nm NIR	Area contraction	40% (1 h)	Yes	[29]
SheathSCH		808 nm NIR	Area contraction	46% (120 s)	Yes	[30]
UPy/GU-gelatin/PNIPAM/ GMA/BTO@Au	Dual thermoresponsive mechanism	37 °C + NIR	Area contraction;	51.7–67.1%;	No	[31]
CNx-IPN	pH- and LCST- triggered deswelling	pH 7.3 to 3.6; 10 °C, 40 °C	Volume contraction	≈40% at pH 7.3 (10 °C); ≈50% at pH 3.6 (40 °C)	Yes	[32]
HCMC/Fe_3_O_4_	pH-triggered deswelling	pH 7.0 to 1.0	Diameter contraction	≈30% (3 h)	Yes	[33]
MA-HA/AA	pH-triggered deswelling	pH 1.2	Diameter contraction	≈15% (24 h)	N/A	[34]
CCS@gel	Ionic crosslinking	pH 7.4 (PBS)	Mass contraction	50% (24 h)	N/A	[35]
α-CD/Azo	Host–guest isomerization	UV/Vis	Volume contraction	124% → 104% (1 h)	Yes	[36]
AAm/TAHA/WP5/MBA		Aqueous diffusion	Area contraction	≈89.2% (10 h)	Yes	[37]
Silk fibroin/gelatin	Protein self-assembly	PBS (37 °C)	Volume contraction	20–40% (72 h)	No	[38]

Notes: LCST, lower critical solution temperature; NIR, near-infrared; UV, ultraviolet; Vis, visible light; PNIPAM, poly(N-isopropylacrylamide); HA, hyaluronic acid; SA, sodium alginate; PVA, poly(vinyl alcohol); PAA, poly(acrylic acid); PEG, polyethylene glycol; GelMA, gelatin methacryloyl; CS, chitosan; GO, graphene oxide; MXene, transition metal carbides/nitrides; PBS, phosphate-buffered saline; TA, tannic acid; PDA, polydopamine; PF127, Pluronic F127. All hydrogel abbreviations are defined at first appearance in the main text. Polymer nomenclature follows standard IUPAC conventions. “N/A” denotes not available or not reported. Contraction data were reported per study-specific metrics; area, volume, diameter, and mass changes are methodologically distinct and not directly comparable across hydrogel systems.

**Table 2:** Comparative overview of different contractile hydrogel systems for wound healing.

Category	Driving Mechanism	Advantages	Limitations	Contraction Characteristics	Potential Applications
Thermoresponsive	LCST-induced chain dehydration and collapse	Fast response; reversible; controllable	Temperature dependence; limited force transmission	Rapid temperature- triggered shrinkage	Acute and chronic wounds
pH-responsive	Protonation/deprotonation- induced charge rearrangement	Self-adaptive; no external trigger	Limited by wound pH variation	pH-dependent network contraction	Chronic and infected wounds
Intermolecular-interaction-driven	Hydrogen bonding, hydrophobic interaction, π–π stacking, coordination, host–guest interactions	Highly tunable; programmable contraction	Slow kinetics; stability concerns	Spontaneous network densification	Sustained mechanical regulation
Solvent/environment-induced	Solvent exchange, water loss, structural rearrangement	Physiological responsiveness; energy-free	Sensitive to hydration conditions	Gradual and sustained contraction	Moist wound; long-term mechanical modulation

**Table 3:** Representative contractile hydrogels for skin wound healing.

Hydrogel System	Responsive Mechanism	Wound Model	Reported Contraction-Related Parameters	Therapeutic Outcomes	Refs
Acute full-thickness skin defects					
PF127-CHO&Gel-CDH@PLGA+ lisinopril	LCST-induced contraction (37 °C)	C57BL/6 mice; splinted full-thickness wound model; 8 mm diameter; 15 days	Qualitative contraction; active wound-edge traction	Wound closure 68% (10 d), 76% (15 d); scar ↓40%; collagen ↑2.5×; M2 polarization ↑	[111]
SA/Gelatin/SNPs	Humidity-driven	SD rats; full-thickness wound model; splint N/A; 10 × 5 mm; 9 days	Shrinkage 16.1%; contraction stress 24 × 103 kPa; traction force >1 N	Healing completed (9 d); angiogenesis ↑; inflammation ↓	[83]
PNIPAM/PVA/ GCCS/Zn^2+^	LCST-induced contraction (37 °C)	BALB/c mice; acute full-thickness wound model; splint N/A; size N/A; 10 days	Shrinkage 52.81%; Wound contraction	Wound closure 97.95%; collagen deposition 52.77% (10 d); VEGF ↑; IL-6 ↓	[115]
WSCA/PNIPAM/MXene	LCST-induced contraction (37 °C)	Kunming mice; full-thickness wound model; splint N/A; size N/A; 14 days	Shrinkage 53.53–58.63% within 15 min (37 °C); force 5.09 kPa	Skin appendage regeneration; TNF-α ↓; IL-10 ↑	[116]
PNIPAM/SA/ GO/CS	Thermo + NIR	Kunming mice; full-thickness wound model; splint N/A; 6 mm diameter; 10 days	Complete closure (7 d)	Complete closure (day 10); CD31 ↑; inflammation ↓; scar reduction	[42]
Infected and diabetic chronic wounds					
HA/PNIPAM/IB-367/Ten-2	LCST-induced contraction (37 °C)	High-fat diet-induced diabetic ICR mice; infected full-thickness wound model;splint N/A; 6 mm diameter; 14 days	Shrinkage 46% (0.5 h) 91% (14 d)	Enhanced angiogenesis (CD31/VEGF ↑), IL-6 ↓, Ki67 ↑, collagen deposition ↑	[117]
PAAm-DN/PNIPAM-DN/Ag-TA/Gelatin	Photothermal (NIR 808 nm)	Rats; splinted infected full-thickness wound model; size N/A; 12 days	Shrinkage 60% (4 min)	Wound closure highest (12 d); IL-6 ↓; collagen ↑; angiogenesis ↑	[118]
PNIPAM/Alginate/MBAA/CaCl_2_/ AHF	LCST-induced contraction (37 °C)	db/db mice; diabetic chronic wound model; splint N/A; 5 mm diameter; 14 days	Shrinkage 77.51% (14 d)	Re-epithelialization ↑; CD206 ↑8.4×; CD31 ↑6.1×; ECM remodeling ↑	[22]
UPy-modified Gelatin/PNIPAM/GMA/BTO@Au	Thermo + NIR	Mice; MRSA-infected full-thickness wound model; splint N/A; 8 mm diameter; 15 days	pore size reduction: 57.0 μm → 21.0 μm within 30 min (37 °C)	Wound closure rate 90%(5 d); MRSA eradication >99%; collagen density ↑238%; hair follicle regeneration ↑; inflammation ↓; TNF-α ↓; VEGF ↑	[31]
PNIPAM/PF127/PEGDA575-Do	LCST-induced contraction (37 °C)	Pig model; skin defect model/SD diabetic rats; MRSA-infected diabetic wound model;splint N/A; 8 mm diameter; 14 days	Pig: Shrinkage 30.46% (5 min) Rat: Shrinkage 97.95% (14 d)	Wound closure rate of 97.95% (14 d); IL-6 ↓; VEGF ↑; Re-epithelialization ↑; angiogenesis ↑; antibacterial effect	[119]
PNIPAM/Alginate/FGF21	LCST-induced contraction (37 °C)	db/db mice; diabetic full-thickness wound model;splint N/A; size N/A; 14 days	Shrinkage ≈ 70% (3 h) Wound traction-induced contraction ratio >15%	Re-epithelialization, collagen deposition, and angiogenesis; abnormal lipid metabolism ↑; ROS damage ↓	[120]
PNIPAM/AAc/ GG/TA	LCST-induced contraction (37 °C)	ICR mice; diabetic full-thickness wound model; splint N/A; 8 mm diameter; 21 days	Mild contraction; porosity change 61.2% → 57.5%	Wound healing 93.8% (21 d); YAP/TAZ- mediated fibroblast activation; inflammation ↓	[121]
Incision repair and scar control					
M-HPMC/PAA	LCST-induced contraction (37 °C)	SD rats; splinted full-thickness wound model; 10 mm diameter; 6 weeks	Tensile strength of 4.72 ± 0.47 MPa	Scar area ↓; collagen deposition ↓ 15%; TGF-β1 ↓; mechanotransduction inhibited	[27]
PNIPAM/SA/OSA/CMC	Photothermal (NIR 808 nm)	SD rats; full-thickness wound model; splint N/A; 1.5 cm in length; 10 days	Scarring reduced, tensile strength increasing by 20.3~31.9%	Complete wound closure (10 d); inflammation ↓; angiogenesis ↑	[29]

Note: LCST, lower critical solution temperature; NIR, near-infrared; UV, ultraviolet; Vis, visible light; PNIPAM, poly(N-isopropylacrylamide); HA, hyaluronic acid; SA, sodium alginate; PVA, poly(vinyl alcohol); PAA, poly(acrylic acid); PEG, polyethylene glycol; GelMA, gelatin methacryloyl; CS, chitosan; AHF, hydroxyl-modified C70 fullerene; OSA, oxidized sodium alginate; CMC, carboxymethyl chitosan; GO, graphene oxide; MXene, transition metal carbides/nitrides; TA, tannic acid; PDA, polydopamine; PF127, Pluronic F127; PAAm, polyacrylamide; DN, double network; GCCS, modified carboxylated chitosan; FGF21, fibroblast growth factor 21; MRSA, methicillin-resistant Staphylococcus aureus; db/db mice: leptin receptor-deficient diabetic mice); SD rats: Sprague-Dawley rats; ROS, reactive oxygen species; ECM, extracellular matrix; CD31, platelet endothelial cell adhesion molecule-1; VEGF, vascular endothelial growth factor; IL-6, interleukin-6; Ki67, proliferation marker Ki-67; TNF-α, tumor necrosis factor-alpha; IL-10, interleukin-10; TGF-β1, transforming growth factor-beta 1; YAP/TAZ, Yes-associated protein/transcriptional coactivator with PDZ-binding motif; M2, alternatively activated macrophages; N/A, not available or not reported. “↑” and “↓” indicate increase and decrease, respectively.

## Data Availability

No new data were created or analyzed in this study. Data sharing is not applicable to this article.
